# Indigenous Community Views of Disability in Canada: Protocol for a Scoping Review

**DOI:** 10.2196/57590

**Published:** 2025-03-10

**Authors:** Andrés Rojas-Cárdenas, Shaun Cleaver, Ivan Sarmiento, Julie Rock, Yan Grenier, Francis Charrier, Rose-Anne Gosselin, Anne Cockcroft, Neil Andersson

**Affiliations:** 1 CIET-PRAM Department of Family Medicine McGill University Montreal, QC Canada; 2 École de readaptation Faculté de médecine et des sciences de la santé Université de Sherbrooke Sherbrooke, QC Canada; 3 Département de psychoéducation et travail social Université du Québec à Trois-Rivières Trois-Rivières Canada; 4 Faculté des sciences sociales École de travail social et de criminologie Université Laval Québec, QC Canada; 5 Centre interdisciplinaire de recherche en réadaptation et intégration social Université Laval Québec, QC Canada; 6 First Nations Human Resources Development Commission of Quebec Kahnawake, QC Canada; 7 Centro de Investigación de Enfermedades Tropicales (CIET) Universidad Autónoma de Guerrero Guerrero Chilpancingo Mexico

**Keywords:** Indigenous health, intercultural dialog, cultural safety, traditional medicine, disability

## Abstract

**Background:**

Indigenous people do not necessarily view disability in the same way as do other groups. Indigenous concepts of disability are connected to their ancestral history, cultural customs, and environmental context. Some Indigenous languages do not contain a word equivalent to disability. Western approaches to disability seldom reflect the voices of Indigenous people.

**Objective:**

The objective of this scoping review is to collate the perspectives, concepts, and understandings of disability in Indigenous communities in Canada and to map the factors that influence social approaches to disability from an Indigenous perspective.

**Methods:**

Following the methodological framework for scoping reviews of Arksey and O’Malley, we will search electronic databases, including PubMed, Scopus, Web of Science, EBSCOhost ProQuest, Autochtonia, and APA PsycINFO. We will search gray literature through the Google search engine, conference abstracts, dissertation databases, government documents, and Indigenous organization websites. We will include quantitative, qualitative, and mixed methods studies in English and French. The included studies will describe Indigenous approaches to disability, as they are understood based on personal, cultural, and historical contexts. Two reviewers will use Covidence software (Cochrane) to remove duplicates, screen articles, record the step-by-step selection process, and extract data from the included articles. We will follow the PRISMA-ScR (Preferred Reporting Items for Systematic Reviews and Meta-Analysis extension for Scoping Reviews) guidelines. We will present the findings in tables, charts, narrative summaries, and through fuzzy cognitive mapping. We will contextualize the literature’s findings by comparing them with the stakeholders in Quebec and provide a discussion to explore potential solutions for the identified factors.

**Results:**

An initial limited search was conducted in January 2024. The study will be conducted in 2025. Publication of the results is expected in late 2025.

**Conclusions:**

We anticipate that the findings from the scoping review will be useful for professionals, researchers, policy makers, and Indigenous communities themselves interested in co-designing and implementing evidence-informed disability programs and services, which will prevent mismatches between the programs and the sociocultural context. We will disseminate the results of this review through workshops with the participating communities, direct engagement with relevant local stakeholders, and through conference presentations and publications in scientific journals.

**Trial Registration:**

OSF Registries osf.io/9rzkx; https://osf.io/9rzkx

**International Registered Report Identifier (IRRID):**

DERR1-10.2196/57590

## Introduction

The way disability is defined affects the types of disability services offered, who uses the services, and the way they work. The traditional biomedical model, despite frequent criticism, continues to influence Western approaches to disability [1,2]. This model views disability as an individual issue, characterized by abnormalities of the body or mind that medical science treats and social services cater for. In this mindset, experts are seen as the professionals responsible for either treating disabled individuals or coordinating services for them. Also focused on individual abnormalities and perhaps more criticized than the biomedical model for treating differences as medical issues and creating population categories, the moral model depicts disability either as a source of stigma and shame or, alternatively, as a sign of strength [3,4].

Social and inclusion models of disability reflect interactions between people with disabilities and attitudinal or environmental barriers that prevent their full and effective participation in society [5]. These approaches go beyond disability as a personal attribute to frame disability in terms of the conditions created by the social environment that cause people to experience barriers to performance in life situations. In the same way, the International Classification of Functioning, Disability, and Health (ICF) reinforces this view by emphasizing that disability results from the interaction between health conditions and contextual factors both environmental and personal [6,7]. The ICF highlights the importance of addressing not only impairments but also the external barriers that limit activity and participation [3,7,8]. Another approach explores not only physical or mental impairments but also the societal norms that define certain characteristics as disabilities. It’s important to examine how social conditions exacerbate these stigmatized characteristics within specific populations [9-11]. A constructive step might be to engage with Indigenous communities to discuss the language and concepts surrounding disability, particularly whether colonialism and its resulting social disadvantages have transformed the social meaning of disability for Indigenous people [12,13].

Literature over the last 2 decades describes Indigenous perspectives and beliefs about disability [12,14-16]. Many Indigenous languages have no word for “disability,” suggesting it is a term produced by western constructs [17], rather than anything negative or based on difference [18,19]. Articles about the intersection of indigeneity and disability highlight the importance of family ties, community networks and spirituality [14,16,18,20-24]. Although clearly different from western views, Indigenous worldviews of disability are not monolithic. Views vary between Indigenous cultures and, within any 1 culture, may not be static but responsive to the recovery of traditions and to adaptations [24,25].

With no published systematic review about Indigenous perspectives of disability from Canada, a review from Australia provides a substantial advance in the field [23]. This examined the understanding of disability within Australian Indigenous communities, highlighting that some disability services are shaped by western norms and assumptions that do not reflect their values. The review also notes that scholarly literature on Indigenous conceptualizations, experiences, and practices of disability remains relatively underdeveloped. Our review explores similar themes within the Canadian context. Our aim is to describe the concepts of First Nations, Métis, and Inuit, the 3 constitutionally recognized Indigenous groups in Canada who comprise approximately 5% of the country’s population [26] and reflect unique cultural, environmental, and historical influences. Our review will focus on Indigenous points of view, summarizing the diverse narratives and choosing not to reproduce damage-centered approaches that contributed to pain and oppression [27].

We describe here the protocol for a scoping review, a starting point of a larger research program. This will compare and combine different knowledge sources to inform partnerships that address knowledge, policy, and implementation gaps for disability support services in Indigenous communities. The broader research initiative will encompass the following knowledge resources: (1) the scoping review detailed in this protocol; (2) perspectives from personnel of community-based initiatives and Indigenous-led organizations; (3) perspectives of Indigenous elders and knowledge keepers; and (4) perspectives from disability service providers, researchers, policy makers and people encountering disabling situations from the communities and their peers. The Weight of Evidence approach [28] uses fuzzy cognitive mapping [29] and will integrate these diverse sources of knowledge.

## Methods

### Study Design

We used the PRISMA-P (Preferred Reporting Items for Systematic Review and Meta-Analysis Protocols) [30] checklist (Multimedia Appendix 1) for drafting the protocol. We will conduct the scoping review according to the guidelines proposed by Arksey and O’Malley [31] and the modifications proposed by Levac et al [32]. The 6-stage process includes identifying the research question, identifying relevant studies, developing a study selection and data extraction method, charting the data, collating, summarizing, and reporting results, and contextualization with stakeholders. We will adapt the final step to function as a contextualization exercise, guided by the weight of evidence approach [28] that contrasts literature findings with contributions from stakeholders. The final report will follow the PRISMA-ScR (Preferred Reporting Items for Systematic Reviews and Meta-analyses extension for Scoping Reviews) [33].

Our team consists of 2 Indigenous researchers with expertise in Indigenous perspectives, psychosocial interventions, and inclusion. Other authors bring significant experience working with Indigenous communities in Canada and Mexico, as well as extensive expertise in working with people with disabilities in Nigeria, Zambia, Canada, and Colombia. We will do the first contextualization in the province of Quebec, as we describe below. We will verbally share discussions with our Indigenous researcher partners in Nunavik and Indigenous community members in the Saguenay-Lac-Saint-Jean region (Mashteuiatsh community) and the team leading the Nisidotam Inclusion Initiative in the Greater Montreal area. This ensures that Indigenous worldviews inform the approach, analysis, and findings, including the discovery and description of key knowledge gaps. We may make changes to the protocol, and we will detail and justify any modifications in the final report.

### Stage 1: Identifying the Research Question

Our research questions are as follows:

1. What does the literature reveal about Indigenous perspectives, concepts, and understandings of disability in Indigenous communities in Canada?
2. What are the key factors that influence disability among Indigenous communities in Canada?

#### Inclusion Criteria

The participants, concept, and context (PCC) framework will guide the inclusion of eligible studies in this scoping review [34].

#### Participants

This review will consider sources that include the First Nations, Inuit, and Métis in Canada as the population of interest. We will include the recognized names of Indigenous peoples as search terms in this review.

#### Concept

This review will consider studies on Indigenous perspectives, concepts, and understandings of disability in Canada, as well as the factors influencing disability among Indigenous people.

#### Context

This review will consider studies or reports conducted in any Canadian province or territory, as well as those with a substantial focus on the Canadian context.

#### Types of Sources

This scoping review will consider peer-reviewed and gray literature. Mixed methods, quantitative, and qualitative studies are eligible for inclusion. The publication date range will be unrestricted.

### Stage 2: Identifying Relevant Studies

We will consider sources that describe or reflect Indigenous perspectives on disability, whether they are published in peer-reviewed journals or as gray literature. The study or report must be located within Canada or have a significant component of the Canadian context.

We will design a structured search strategy for the electronic databases PubMed, Scopus, Web of Science, EBSCOhost (Bibliography of Indigenous Peoples in North America), ProQuest (Canadian Business and Current Affairs Database), Autochtonia, and APA PsycINFO to identify relevant published studies. We will develop the strategy using Boolean operators, filters, and truncation for each database. We will search for relevant articles using a mixture of search terms and keywords. We will consider adapting the filter developed by the University of Alberta to retrieve studies related to Indigenous people [35]. The research team will draft and refine a search strategy with the support of a professional librarian. We will pilot the search strategy to check the appropriateness of the keywords and databases. As an example, Multimedia Appendix 2 presents the search strategy for Scopus database.

The search for potentially relevant documents in the gray literature will follow 4 different searching strategies [36]: (1) search gray literature databases such as the Canadian Research Index and the Indigenous Studies Portal at the University of Saskatchewan; (2) use customized Google search engines to examine the first ten pages of results. Combine keywords using simple Boolean operators or hand search relevant subsections of sites; (3) Target Indigenous associations and websites for relevant information; and (4) Consult with experts proficient in research synthesis and aware of relevant documents to gather additional insights. We will contact both Indigenous and non-Indigenous scholars to include multiple sources and ensure our search is as inclusive as possible.

We will hand-search the references for the included articles to identify any additional relevant articles. We will limit the search to articles published in English or French. We will not apply restrictions based on the year of publication or study design.

To stay updated of current work in the field, our scoping analysis methodology will allow us to continually circle back to take newer articles through the screening process and potentially include them in our analysis.

### Stage 3: Study Selection

We will export the list of references into Covidence software (Veritas Health Innovation) [37] to conduct title or abstract and full-text screening, first using the software to remove duplicates. Two independent researchers will screen study titles and abstracts against the inclusion and exclusion criteria to identify potentially relevant articles for full-text review. Two reviewers will then conduct a full-text review to confirm the final selection of articles. We will resolve disagreements by consensus or by consulting a third reviewer. A PRISMA (Preferred Reporting Items for Systematic Reviews and Meta-analyses) flow chart will show the study selection procedure.

### Stage 4: Charting the Data

Two reviewers will use the Covidence data charting framework to extract the relevant data from the included articles. They will pilot the draft data extraction sheet on a random sample of 5 articles. They will use a free use application to chart the data. The first part of the data extracted will include the authors, study title, source or journal, publication type, year of publication, objectives of the study, study design, geographic location, Indigenous group, description of participants, language, findings on perspectives, concepts, understandings, and terminology used about disability. The second part will list all the factors that influence disability from the Indigenous perspective and their relationships. We will present a 3-column edge list, a tabular format to represent relationships in a fuzzy cognitive map. The columns will include causes (originating node), outcomes (landing node), and the sign of the relationship (–1 or +1) [29]. Additional columns will indicate supporting evidence for the relationship and corresponding reference. We will use 1 row for each relationship. If the evidence is quantitative, we will include relationships that are significant at the 95% confidence level. If the evidence is qualitative, we will include quotes, arguments or texts supporting the relationship. We will adjust and refine the data extraction form as needed throughout the data extraction process and document any modifications in the review report. Data extractors will resolve disagreements through discussion.

### Stage 5: Collating, Summarizing, and Reporting Results

We will present the results of the review as a qualitative description, incorporating tables, figures, and maps where appropriate. We will present the findings of the scoping review in a fuzzy cognitive map to illustrate the perspectives, concepts and understandings of disability and the factors that influence disability from an Indigenous perspective.

Fuzzy cognitive mapping visually represents knowledge, helping clarify the complex factors that influence outcomes or decisions [38,39]. These maps show assumed causal relationships that can be based on data or unwritten knowledge [40] between concepts or factors (nodes) and the outcomes, connected by arrows or edges [38,39,41]. The source of knowledge assigns different values to the edges, indicating the direction of the causal relationships and quantifying the strength of their influence on the outcome [28,38,39,41,42].

We will create a map for each article included in the review. We will identify whether the influence of a reported factor is positive or negative (+1 or –1) and depict it in a 3-column edge table as described above. If the study shows that an increase in one factor leads to an increase in another factor, we will assign a positive relationship (+1). If it shows a decrease in the second factor, we will assign a negative relationship (–1) [42].

After creating an individual table for each included study, we will calculate the fuzzy transitive closure for each study using the open access software CIETmap (V.2.2) to determine the strength of influence one factor has on others through direct or indirect relationships [43].

We will use Harris’ discourse analysis approach to weigh each relationship based on its relative frequency across all the transitive closure maps [44,45]. Factors appearing in multiple maps will be weighted as having a stronger causal influence than those appearing in only 1 or 2 studies [41,45]. By dividing each relationship’s occurrence by the highest frequency, we will obtain values between 0 and 1, where values closer to 1 indicate more influence. We will create a composite map of all factors and relationships identified in the scoping review, with relationship weights based on their relative frequency [45]. Multimedia Appendix 3 illustrates an example of a fuzzy cognitive map.

We anticipate refining and expanding the data presentation approach as the nature of the available literature becomes known. We will highlight areas where evidence is lacking and make recommendations for decision-making, practice or further research.

We will report the review methods and findings according to the PRISMA-ScR guidelines. We will disseminate the results of the scoping review in a peer-reviewed publication and present and discuss them in relevant forums, workshops, conferences, and community spaces. Potential limitations of the review include that we may miss studies reported in Indigenous languages and that some local knowledge may not be accessible despite best efforts to search the gray literature.

### Stage 6: Contextualization

We will contextualize with stakeholders in the final step of the scoping review [31,32], which is part of a broader participatory research project on disability among Indigenous peoples of Canada. We will adapt the “weight of evidence” approach [28] to contrast and combine the synthesized evidence from the literature with the experiential knowledge of stakeholders, including Indigenous scholars, elders, knowledge keepers, cultural advisors, individuals with disabilities from the communities and their peers, as well as members of community-based and regional organizations representing people with disabilities or First Nations and Inuit in Quebec. We will invite them to share their perspectives, concepts, and understandings of disability in Indigenous communities, and to create maps of the factors that influence disability from an Indigenous perspective.

We will adapt the fuzzy cognitive mapping protocol recommended by Andersson and Silver [39]. A facilitator and a notetaker will support each stakeholder mapping session.

After the stakeholders create their own maps, they will compare them with the composite map from the scoping review. We will combine the scoping review map with the users’ maps to update the literature with stakeholder perspectives. The scoping review and the stakeholder maps will serve as the basis for engaging them to explore solutions for the identified issues.

## Results

The preliminary database search was conducted in January 2024. The study is scheduled for 2025, and its results will be published in open-access, peer-reviewed journals by the end of 2025.

## Discussion

### Principal Findings

The proposed scoping review will identify and map evidence on Indigenous concepts, perspectives, and understandings of disability as well as the key factors that influence disability in Canada. This review offers space for perspectives outside Western paradigms, facilitating a subsequent intercultural dialogue. This dialogue allows stakeholders with different cultural backgrounds to engage in respectful, dynamic communication to address a concern [46], in this case, the conceptualization of disability in Indigenous communities. The review might encourage a more inclusive, comprehensive, and culturally safe understanding of disability. It will contextualize the existing literature in the rich heritage of Indigenous Peoples in Quebec, Canada, including their perspective of disability and the implications of this for disability policy in this province. It will serve as a prototype for contextualizing the literature in the specific belief systems of Indigenous groups in other provinces and beyond Canada.

To our knowledge, no previous literature review has covered this topic in Canada. However, a review published by Australian authors significantly advances the understanding of disability services and disability conceptualization within Indigenous communities.

### Strengths and Limitations

This scoping review has several strengths. First, we will include gray literature. By doing so, we will add valuable insights and ensure we don’t miss findings and diverse perspectives that may not be found in peer-reviewed academic sources. Second, by contrasting and integrating evidence from the literature with the knowledge of Indigenous communities and people with disabilities through fuzzy cognitive maps, the scoping review will provide a thorough overview. Third, the participatory nature of the exercise will engage all stakeholders in interrogation of the literature and the codevelopment of a uniquely provincial and inclusive perspective. In this important sense, participation in the review process can be a first step in the intercultural dialogue.

The scoping review will have certain limitations. Some studies in Indigenous languages might be missed, and certain local knowledge may remain inaccessible, despite diligent attempts to include gray literature. The review will not include a quality assessment of the studies, which may introduce a risk of bias. We will provide a description of the methodologies and different criteria used to facilitate the contextualization of the findings. Potential limitations could include challenges in language use during fuzzy cognitive mapping for stakeholders, operator bias (influence of facilitators), as well as issues with coding and weighting [29]. To address this, we will train facilitators in protocols to reduce these recognized weaknesses. We are aware that the inclusion of multiple interpretations, factors, and varying levels of methodological rigor across different groups could decrease the precision of results.

### Future Directions

By mapping the literature and identifying knowledge gaps, this scoping review will be a first step toward identifying research priorities and developing effective policies and interventions for Indigenous groups with disabilities. Future directions include repetition of the contextualization exercise in other Canadian provinces, generating provincially specific reviews that take account of Indigenous views in that province and the provincial agencies concerned with Indigenous people living with disability.

In addition to scientific publication and conference presentations, we will share the findings through workshops with participating communities and civil society organizations working in disability in Quebec.

## Appendix

Multimedia Appendix 1 PRISMA-P (Preferred Reporting Items for Systematic Reviews and Meta-Analyses Protocols) 2015 checklist.

Multimedia Appendix 2 Search strategy for scopus.

Multimedia Appendix 3 Example of a fuzzy cognitive map.

## Abbreviations

ICF: International Classification of Functioning, Disability, and Health
PCC: participants, concept, and context
PRISMA: Preferred Reporting Items for Systematic Review and Meta-Analysis
PRISMA-P: Preferred Reporting Items for Systematic Review and Meta-Analysis Protocols
PRISMA-ScR: Preferred Reporting Items for Systematic Review and Meta-Analysis extension for Scoping Reviews
