# Supplementary material for: Indigenous Community Views of Disability in Canada: Protocol for a Scoping Review
Source: JMIR Res Protoc. 2025 Mar 10;14:e57590. doi: 10.2196/57590 (PMC11933751; doi:10.2196/57590)
Supplement: Multimedia Appendix 2 [file resprot_v14i1e57590_app2.docx]

**Appendix 1: Search strategy for Scopus.**

( ( TITLE-ABS-KEY ( "Disabled Persons" OR disabil* OR "persons with disabilit*" OR "people with disabilit*" OR handicap* OR "special need*" OR "care need*" OR impairment OR impaired OR "People living disabling situations" OR "encountering disabling situations" ) ) )

AND

( ( TITLE-ABS-KEY ( perspecti* OR concept* OR construct* OR belie* OR understand* OR definition ) ) )

AND

( ( TITLE-ABS-KEY ( native* OR indigenous OR "First Nation*" OR metis OR inuk OR inuit OR eskimo* OR "American Indian*" OR aboriginal* OR amerindian* OR autochtone* OR athapaskan OR saulteaux OR wakashan OR cree OR dene OR inuit OR inuk OR inuvialuit* OR haida OR ktunaxa OR tsimshian OR gitsxan OR "Nisga&apos;a" OR haisla OR heiltsuk OR oweenkeno OR "Kwakwaka&apos;wakw" OR "Nuu chahnulth" OR "Tsilhqot&apos;in" OR dakelh OR "Wet&apos;suwet&apos;en" OR sekani OR "dunneza" OR dene OR tahltan OR kaska OR tagish OR tutchone OR nuxalk OR salish OR "stl&apos;atlimc" OR "nlaka&apos;pamux" OR okanagan OR "Secwepmc" OR tlingit OR anishinaabe OR blackfoot OR nakoda OR tasttine OR "Tsuu T&apos;ina" OR "Tsuut&apos;ina" OR "Gwich&apos;in" OR ( han AND NOT chinese ) OR tagish OR tutchone OR algonquin OR nipissing OR ojibwa OR potawatomi OR innu OR maliseet OR "Mi&apos;kmaq" OR micmac OR passamaquoddy OR haudenosaunee OR cayuga OR mohawk OR oneida OR onodaga OR seneca OR tuscarora OR wyandot OR metis OR "red road" OR "on reserve" OR "off-reserve" OR "Original People*" OR ( urban W/3 ( indian* OR native* OR aboriginal* ) ) OR "country food*" OR "residential school*" ) ) )

AND

( ( TITLE-ABS-KEY ( canad* OR "British Columbia" OR "Colombie Britannique" OR alberta OR saskatchewan OR manitoba OR ontario OR quebec OR "Nova Scotia" OR "Nouvelle-Ecosse" OR "New Brunswick" OR "Nouveau-Brunswick" OR newfoundland OR "Terre-Neuve" OR labrador OR "Newfoundland And Labrador" OR "Terre-Neuve-Et-Labrador" OR "Prince Edward Island" OR "Ile-Du-Prince-Edouard" OR yukon OR nwt OR "Northwest Territories" OR "Territoires Du Nord-Ouest" OR nunavut OR nunavik OR nunatsiavut OR nunatukavut OR federal OR provincial OR municipal ) ) )
