# Supplementary material for: Indigenous Community Views of Disability in Canada: Protocol for a Scoping Review
Source: JMIR Res Protoc. 2025 Mar 10;14:e57590. doi: 10.2196/57590 (PMC11933751; doi:10.2196/57590)

**Appendix 2: Example of a fuzzy cognitive map.**

The map includes concepts, or nodes, connected by arrows. Arrows start from causes and lead to their outcomes. Each weight represents the strength of the relationship between concepts. A weight of 0 indicates no causal link. Negative weights suggest a causal decrease in the outcome, while positive weights indicate a causal increase.


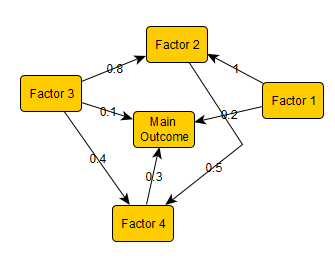

Supplement: Multimedia Appendix 3 [file resprot_v14i1e57590_app3.docx]
